# Pregnancy Is Associated with Decreased Cardiac Proteasome Activity and Oxidative Stress in Mice

**DOI:** 10.1371/journal.pone.0048601

**Published:** 2012-11-15

**Authors:** Andrea Iorga, Shannamar Dewey, Rod Partow-Navid, Aldrin V. Gomes, Mansoureh Eghbali

**Affiliations:** 1 Department of Anesthesiology, Division of Molecular Medicine, David Geffen School of Medicine at University of California Los Angeles, Los Angeles, California, United States of America; 2 Department of Neurobiology, Physiology and Behavior, University of California Davis, Davis, California, United States of America; University of Cincinnati, United States of America

## Abstract

During pregnancy, the heart develops physiological hypertrophy. Proteasomal degradation has been shown to be altered in various models of pathological cardiac hypertrophy. Since the molecular signature of pregnancy-induced heart hypertrophy differs significantly from that of pathological heart hypertrophy, we investigated whether the cardiac proteasomal proteolytic pathway is affected by pregnancy in mice. We measured the proteasome activity, expression of proteasome subunits, ubiquitination levels and reactive oxygen production in the hearts of four groups of female mice: i) non pregnant (NP) at diestrus stage, ii) late pregnant (LP), iii) one day post-partum (PP1) and iv) 7 days post-partum (PP7). The activities of the 26 S proteasome subunits β1 (caspase-like), and β2 (trypsin-like) were significantly decreased in LP (β1∶83.26±1.96%; β2∶74.74±1.7%, normalized to NP) whereas β5 (chymotrypsin-like) activity was not altered by pregnancy but significantly decreased 1 day post-partum. Interestingly, all three proteolytic activities of the proteasome were restored to normal levels 7 days post-partum. The decrease in proteasome activity in LP was not due to the surge of estrogen as estrogen treatment of ovariectomized mice did not alter the 26 S proteasome activity. The transcript and protein levels of RPN2 and RPT4 (subunits of 19 S), β2 and α7 (subunits of 20 S) as well as PA28α and β5i (protein only) were not significantly different among the four groups. High resolution confocal microscopy revealed that nuclear localization of both core (20S) and RPT4 in LP is increased ∼2-fold and is fully reversed in PP7. Pregnancy was also associated with decreased production of reactive oxygen species and ubiquitinated protein levels, while the de-ubiquitination activity was not altered by pregnancy or parturition. These results indicate that late pregnancy is associated with decreased ubiquitin-proteasome proteolytic activity and oxidative stress.

## Introduction

Cardiac hypertrophy, defined as an enlargement of the ventricles and cardiomyocytes, can be adaptive or maladaptive, and usually occurs in response to hemodynamic stress from volume or pressure overload. Sustained pressure overload leads to concentric hypertrophy, which is characterized by increased wall thickness without a concomitant chamber enlargement. However, in response to normal exercise or pregnancy, physiological or eccentric hypertrophy develops [1], which is characterized by an increase in cardiac pumping ability and muscle mass. Volume overload-induced hypertrophy is characterized by a proportional enlargement of the chamber size and the wall thickness [2] and is reversible without aberrant effects on cardiac function [3], [4], [5]. In these aspects, pregnancy- and exercise-induced hypertrophies are similar. However, pregnancy is also accompanied by acute changes in the mother’s hormonal environment, and unlike exercise, the force demand placed on the heart is continuous as opposed to sporadic.

The ubiquitin-proteasome system (UPS) is the major pathway for protein degradation in the heart to remove damaged and misfolded proteins [6]. Regulation of proteasome function can occur through the association of the core 20 S proteasomal complex with different regulatory complexes such as 19 S or 11 S that affect proteasomal assembly and activity [6], [7], [8]. In general, the covalent binding of multiple ubiquitin molecules to the target protein dictates its degradation by the 26 S proteasome [9]. Following attachment of ubiquitin molecules to a target protein, the 19 S regulatory subunits recognize the polyubiquitin tags and transfer the protein substrate to the inner pore of the 20 S catalytic core where the polypeptide is degraded [10]. Proteasome dysfunction in the heart leads to accumulation of abnormal, damaged and misfolded proteins [11].

Altered ubiquitin-proteasome system regulation has been reported in different types of cardiac hypertrophy and myopathy [6], [12]. However, the precise role of the UPS in physiological heart hypertrophy during pregnancy is not yet known. To investigate the role of the UPS in the murine heart during pregnancy, we measured proteasome activity, proteasome subunit expression and subcellular distribution, ubiquitination and de-ubiquitination levels, as well as reactive oxygen production in four groups of female mouse hearts: i) non pregnant (NP) at diestrus stage, ii) late pregnant (LP), iii) one day post-partum (PP1) and iv) 7 days post-partum (PP7). We found that pregnancy is associated with decreased proteasome activity, protein ubiquitination, and oxidative stress.

## Experimental Procedures

### Animals and Treatment

Young adult female (3–4 months) mice (C57BL/6) in non pregnant (NP, at diestrus stage), late pregnant (LP, day 20 of pregnancy), 1 day post-partum (PP1), 7 days post-partum (PP7) as well as ovarictomized (OVX) mice were used. OVX mice were treated with a single subcutaneous 10-day continuous release 17β-estradiol (E2) pellet (0.012 mg/pellet, Innovative Research of America, Sarasota, FL), or placebo pellets (containing 5 compounds: cholesterol, lactose, cellulose, phosphates and cerates) as vehicle for E2. This study was carried out in strict accordance with the recommendations in the Guide for the Care and Use of Laboratory Animals of the National Institutes of Health. The protocol received approval from the Division of Laboratory Animal Medicine at the University of California, Los Angeles (Protocol Number: 2003-111-13).

### Real Time PCR

Total RNA was isolated from hearts using Trizol (Invitrogen) and reverse transcribed with gene specific primers using the Omniscript RT kit (Qiagen). Controls were: (1) the reaction without reverse transcriptase and (2) H_2_O instead of cDNA. The primer sequences used were as follows: RPN2 sense 5′-CAG CTC TCA TCA TGA TCC AAC AG-3′ and anti-sense 5′-ACA GGT AGG AGT ATA AGC CAA TG-3′, RPT4 sense 5′-TGA TCA TGG CTA CAA ACA GAC CA-3′ and anti-sense 5′- CTC AGG TCT GCT CCA TTA AAG C -3′, β2 sense 5′- AGC CAA GAA GCT AGT GAG TGA G-3′and anti-sense 5′- TAT CCA ACC ACC CAC AGC ACC -3′, α7 sense 5′-AAA CAT CGA ACT TGC CGT CAT GA-3′ and anti-sense 5′-GGC CCA CAG CAC CGA GGC T-3′, and PA28α sense 5′-GGC CAC ACT GAG GGT CCA TC-3′ and anti-sense 5′-ACA CAG GTC TTC ACG GAA CAC A-3′. GAPDH transcript levels were used as an internal control.

### Western Blot

Whole heart cell lysates were prepared by homogenizing the hearts in: 50 mM Tris (pH 7.5), 1 mM EDTA, 5 mM MgCl_2_, 150 mM NaCl, 1 mM DTT supplemented with Phosphatase and Protease Inhibitor cocktails (Roche). The samples were then centrifuged at 12,000 g for 10 min and the supernatants were collected. The protein concentrations were measured and 100 µg of protein was treated with SDS/DTT loading buffer prior to gel electrophoresis. The blots were probed with anti-RPN2, -RPT4 -α7, PA28α and –β5i (Enzo Life Sciences, 1∶500) and with anti-mono- and polyubiquitinated conjugates (Enzo Life Sciences, clone FK2, 1∶1000). Quantification of protein levels was achieved using the Metamorph software for protein levels of the proteasome subunits. For the quantification of ubiquitinated protein levels, 100 µg of protein was subjected to standard Western Blotting procedure and immunolabeled with anti-mono- and polyubiquitinated antibodies. The fluorescence intensity of the entire lane was assessed in each group using ImageJ software and average fluorescence intensities were normalized to NP levels.

### Isolation of Cardiomyocytes

The hearts were quickly removed and perfused through the aorta with the following solutions: (i) Ca^2+^-free Tyrode solution containing (in mM): 130 NaCl, 5.4 KCl, 1 MgCl_2_, 0.33 NaH_2_PO_4_, 10 HEPES, 5.5 glucose (pH adjusted to 7.35–7.37 with NaOH) for 5 minutes, (ii) Ca^2+^-free Tyrode solution containing 160.4 U/ml Collagenase Type II (Worthington) and 0.45 U/ml Protease Type XIV (Sigma) for ∼15 min; and (iii) Krebs solution containing (in mM): 100 K-glutamate, 10 K-aspartate, 25 KCl, 10 KH_2_PO_4_, 2 MgSO_4_, 20 taurine, 5 creatine base, 0.5 EGTA, 5 HEPES, 20 glucose (pH adjusted to 7.2 with KOH) for 5 minutes. The solutions were oxygenated with 5% CO_2_ and 95% O_2_ prior to use and were maintained at 37±1°C.

### Immunocytochemistry and Imaging

Freshly isolated cardiomyocytes were fixed in cold acetone for 10 min at −20°C. The isolated cells were incubated with 10% normal goat serum (NGS) to block the background and were then stained with anti-core and anti-RPT4 (Enzo Life Sciences, 1∶200) primary antibodies in 1% NGS and 0.2% Triton X-100 in PBS at 4°C overnight. Cells were incubated with Alexa 488 goat anti-rabbit or Alexa 568 goat anti-mouse secondary antibodies. Images were acquired at 0.0575 nm per pixel with a confocal microscope (Olympus Fluoview). For dihydroethidium (DHE, Invitrogen) staining, whole hearts were excised, washed thoroughly with ice-cold PBS and frozen in O.C.T. compound. Fresh 6 µm sections were cut with a cryostat then incubated with 10 µM DHE in Krebs-HEPES buffer (containing in mM: 99 NaCl, 4.69 KCl, 25 NaHCO_3_, 1.03 KH_2_PO_4_, 5.6 D-Glucose, 20 Na-HEPES, 2.5 CaCl_2_ and 1.2 MgSO_4_) for 1 hr and 15 min in the dark at room temperature. The sections were then washed 3 times for 1.5 hrs in the dark with Krebs-HEPES buffer, mounted with Prolong Antifade Reagent (Invitrogen) and visualized with a confocal microscope (Olympus Fluoview).

### Proteasome Activity Assay

Heart cell lysates were prepared by homogenizing the hearts in: 50 mM Tris, 1 mM EDTA, 5 mM MgCl_2_, 150 mM NaCl, 1 mM DTT, pH 7.5. The samples were then centrifuged at 12,000 g for 10 min and the supernatants were collected. Proteasome activity of heart homogenates (20 µg/sample) was measured with fluorescent substrates of Z-LLE-AMC (β1), Boc-LSTR-AMC (β2) and Suc-LLVY-AMC (β5) as previously described [13], [14]. The proteasome activity was measured in the presence and absence of proteasome inhibitors (40 µM Z-Pro-Nle-Asp-CHO for β1, 40 µM epoxomicin for β2, and 20 µM epoxomicin for β5). Assays were carried out in a total volume of 100 µl. The ATP-dependent 26 S proteasome activities were measured in the presence of 50 mM Tris, 1 mM EDTA, 150 mM NaCl, 10 mM MgCl_2_, 0.1 mM ATP, pH 7.5. The ATP-independent 20 S proteolytic activity for β5 was carried out in 25 mM HEPES (pH 7.5), 0.5 mM EDTA, and 0.03% SDS. The buffer composition was 25 mM HEPES (pH 7.5), 0.5 mM EDTA, 0.05% Nonidet P-40, and 0.001% SDS for β1 and β5 20S activity measurements. These buffers used for 20 S proteasome activity were previously found to be optimal for proteasome activity in lysates from mouse heart tissue [13], [14]. Each assay was conducted in the absence and presence of a specific proteasome inhibitor ((40 µM Z-Pro-Nle-Asp-H for β1, 60 µM epoxomicin for β2 and 20 µM epoxomicin for β5) to determine proteasome-specific activity. Released AMC was measured using a Fluoroskan Ascent fluorometer (Thermo Electron) at an excitation wavelength of 390 nm and an emission wavelength of 460 nm.

### De-ubiquitination Assay

De-ubiquitination activity was determined using 5 µg of protein in 50 mM Tris, 150 mM NaCl, 1 mM EDTA, 5 mM MgCl_2_, 2 mM DTT, pH 7.5. All assays were carried out in a final volume of 100 µl. The reaction was initiated by adding 400nM ubiquitin-AMC (Enzo Life Sciences). Each assay was conducted in the absence and presence of a de-ubiquination inhibitor (10 mM N-ethylmaleimide (NEM)) to determine de-ubiquitination-specific activity. Released AMC was measured using a Thermo Fluoroskan Ascent fluorometer (Thermo Electron) at an excitation wavelength of 390 nm and an emission wavelength of 460 nm.

### ELISA Assay

1µg of protein lysate was bound overnight at 4°C on a 96 well-ELISA plate, washed 4 times, and blocked with BSA. 100 µl of FK1 (1∶1000 dilution) detection antibody (which only recognizes polyubiquitinated proteins and not monoubiquitinated or free ubiquitin) was added for 1 h, washed, and an anti-mouse HRP-conjugated secondary antibody was added for 1 hr. After washing the secondary antibody, 100 µl of Sureblue tetramethylbenzidine substrate (KPL Inc.) was added and incubated for 15 min. The reaction was stopped using 1 M HCl and absorbance was measured at 450 nm. Positive (pentaubiquitin chains, Enzo Life Sciences) and negative controls (BSA) were used to validate the assay.

### Statistics

One-way ANOVA using SPSS SigmaStat 3.0 was used for statistical analysis. *P* values<0.05 were considered significant. Values are mean ± SEM.

## Results

### Proteasome Activity of 26S is Decreased in LP

**Figure 1:**
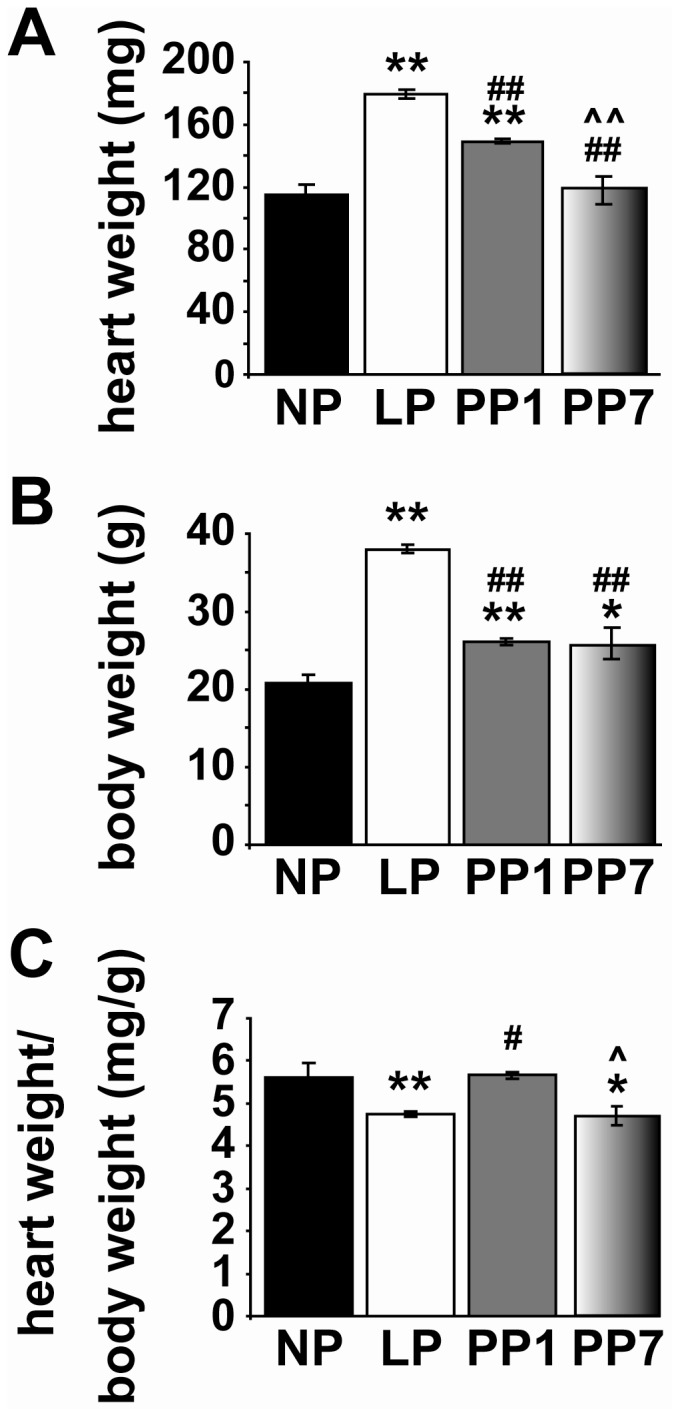
The increased in heart weight of LP mice is reversed post-partum. Heart weights (HW, A), body weights (BW, B) and HW/BW (C) in non-pregnant (NP, black bars, n = 8), late pregnant (LP, white bars, n = 11), one day post-partum (PP1, grey bars, n = 6) and seven days post-partum (PP7, shaded bars, n = 5) mice. Values are mean ± SEM, ^*^p<0.05 and ^**^p<0.001 vs. NP, ^#^p<0.05 and ^##^p<0.001 vs. LP, ^∧^p<0.05 and ^∧∧^p<0.001 vs. PP1.

During pregnancy, the heart develops physiological hypertrophy as a result of the natural volume overload (Fig. 1). However, the ratio of the heart weight to body weight decreases in LP due to a significant increase in body weight at the end of pregnancy as we reported previously [15]. Interestingly, the heart weight is reversed partially one day post partum (PP1) and fully one week post partum (PP7).

**Figure 2:**
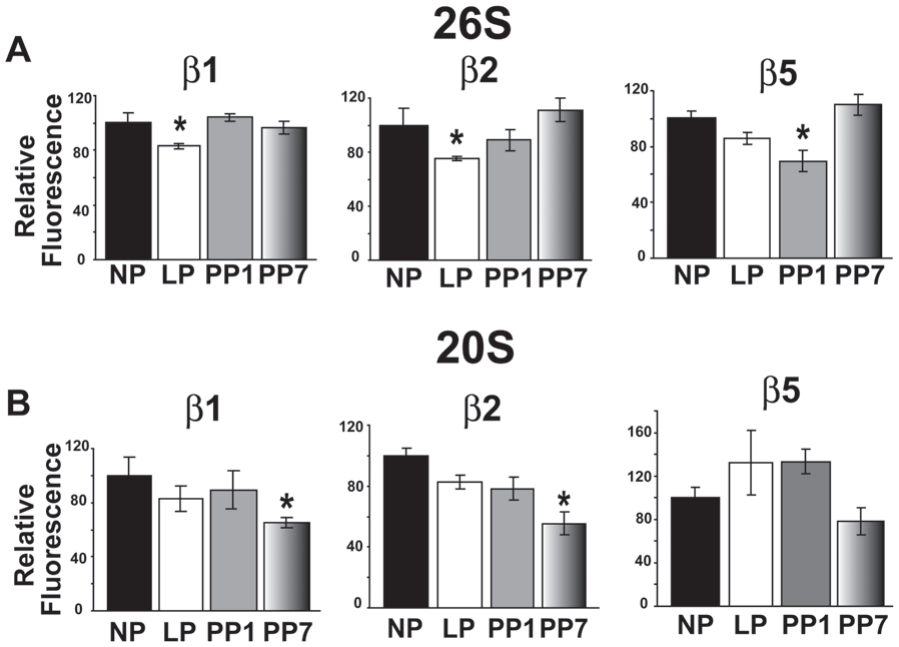
Proteasome activity of 26 S, but not 20 S is reduced in late pregnancy. Activity of different proteasomal beta subunits of the 26 S (A) and 20 S (B) was measured after initiating the reaction with: Z-LLE-AMC (β1), Boc-LSTR-AMC (β2) and Suc-LLVY-AMC (β5) for non pregnant (NP, black bars), late pregnant (LP, white bars), one day post-partum (PP1, grey bars) and seven days post-partum (PP7, shaded bars). The fluorescence values in arbitrary units are represented as mean ± SEM *p<0.05 vs. NP (n = 4 mice per group) and are normalized to NP levels. The raw proteasome activity values for the NP group are as follows (in nmol/min/mg protein): for the 26 S ATP-dependent activities, β1 was 0.11±0.01, β2 was 0.04±0.01 and β5 was 0.16±0.01, while for the 20 S activity, β1 was 0.21±0.03, β2 was 0.15±0.02 and β5 was 0.12±0.01.

To examine whether the proteasome function is altered in the heart in late pregnancy, we measured the proteasome activity of total 26 S ATP-dependent as well as the 20 S ATP-independent proteasome. Regarding 26 S, the relative caspase-like catalytic β1 subunit activity was decreased from 100±6.82% in NP to 83.26±1.96% in late pregnancy, and was restored fully even one day post-partum (Fig. 2). The trypsin-like β2 subunit activity was also decreased in late pregnancy from 100±13.72% in NP to 74.74±1.7% in LP, and was only fully restored in PP7 (111.56±8.7%). Interestingly, the chymotrypsin-like β5 activity of the proteasome was significantly decreased 1 day post-partum (from 100±4.7% in NP to 69.62±4.5% in PP1). All three proteolytic activities of the 26 S proteasome were fully restored to normal levels 7 days after parturition (Fig. 2). Unlike 26 S, the proteasome activity of all three subunits of 20 S was not altered in LP (Fig. 2). However, the activity of β1 and β2 subunits was significantly lower 7 days after parturition (from 100±13.72% in NP to 64.69±3.89% in PP7 and from 100±13.35% in NP to 55.41±4.63% in PP7, respectively (Fig. 2).

### Proteasome Activity is not Regulated by Estrogen Therapy

**Figure 3:**
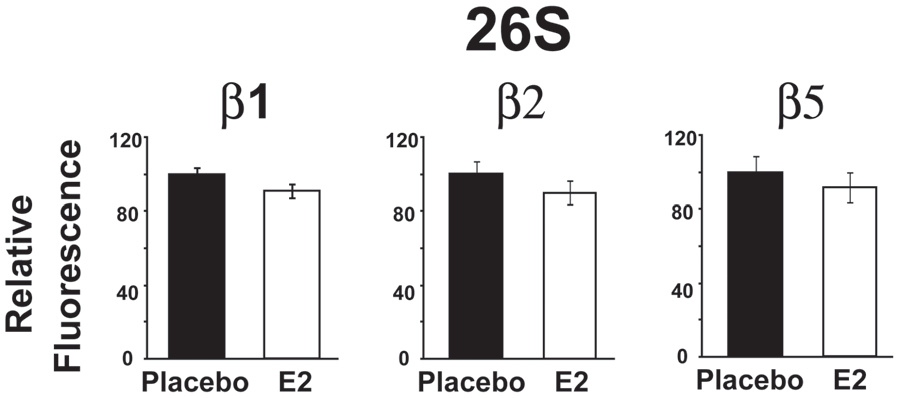
Proteasome activity of the 26S is unaffected by estrogen treatment. Activity of the different proteasomal beta subunits of the 26 S were measured after initiating the reaction with: Z-LLE-AMC (β1), Boc-LSTR-AMC (β2) and Suc-LLVY-AMC (β5) in ovariectomized female mice treated with placebo (Placebo, black bars) or with 17 β-estradiol for 10 days (E2, white bars). The fluorescence values in arbitrary units are normalized to Placebo levels and represented as mean ± SEM (n = 4 mice per group).

The levels of estrogen increases steadily during pregnancy and reach their maximum levels at the end of pregnancy. To elucidate whether decreased proteasome activity at the end of pregnancy was a result of the surge of estrogen, ovariectomized (OVX) mice were treated with E2 or placebo using continuous release pellets for 10 days. The 26 S ATP-dependent proteasome activity was not affected by estrogen treatment, as the activities of the three subunits were not significantly different between E2 and placebo groups (Fig. 3). Therefore, the reduced proteasome activity in late pregnancy could not be explained by the rise of estrogen levels at the end of pregnancy.

### The Transcript and Protein Levels of Proteasome Subunits are not Modified by Pregnancy

**Figure 4:**
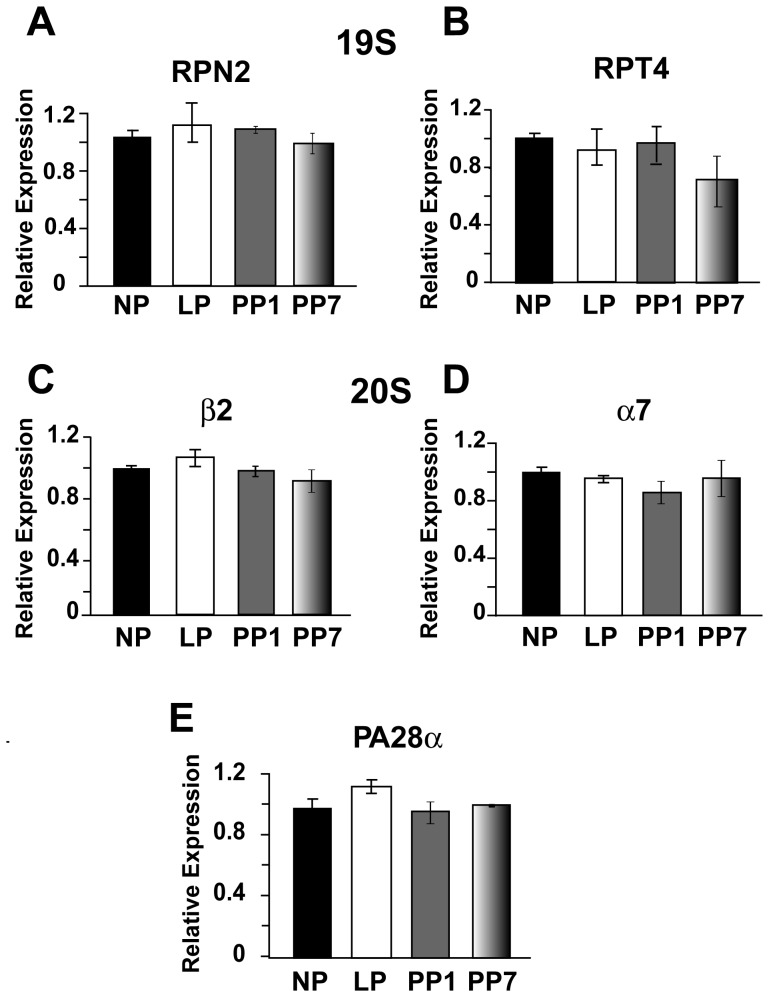
Transcript levels of proteasome 19S and 20S subunits, as well as the regulatory subunit PA28α, are not modified in late pregnancy. Relative transcript expression of the cardiac proteasome measured by Real-Time qPCR in non pregnant (NP, black bars), in late pregnancy (LP, white bars), 1 day post-partum (PP1, grey bars) and 7 days post-partum (PP7, shaded bars) for RPN2 and RPT4, which are subunits of 19 S (A–B), β2 and α7, which are subunits of 20 S (C–D) and the proteasome regulatory subunit PA28α (E). GAPDH was used as the internal reference gene (data not shown). Values are mean ± SEM as normalized to NP (n = 3–5 per group).

**Figure 5:**
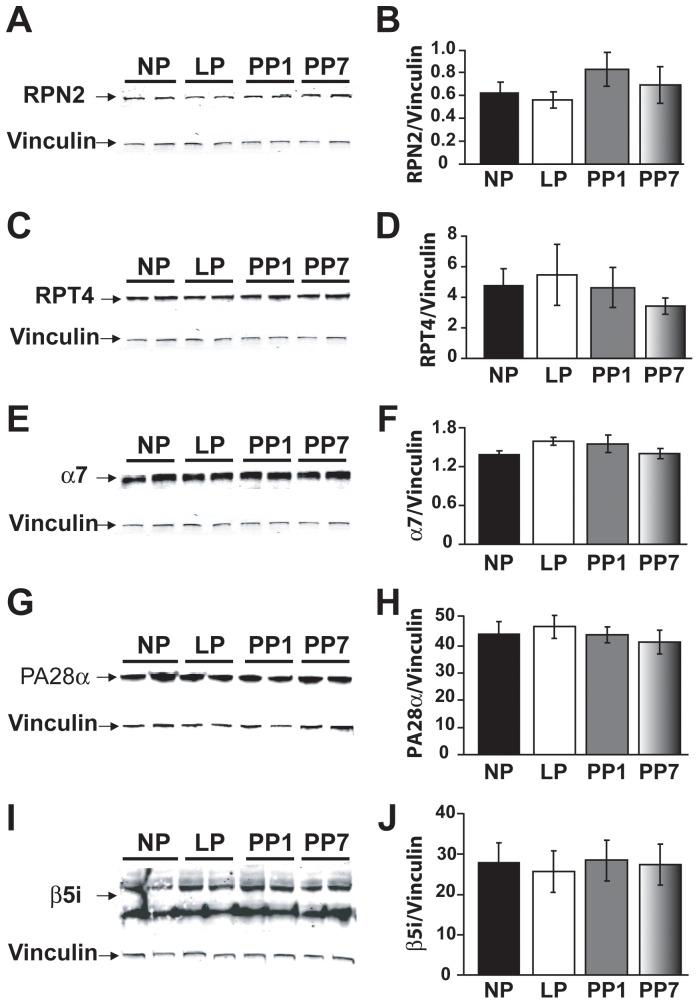
Protein levels of RPT4, RPN2, α7, PA28α and β5i are unaffected by pregnancy. Immunoblotting of whole heart lysates (100 µg) from non pregnant (NP, black bars), late pregnant (LP, white bars), 1 day post-partum (PP1, grey bars) and 7 days post-partum (PP7, shaded bars) with anti-RPN2 (A–B), -RPT4 (C–D), -α7 (E–F), -PA28α (G–H) and -β5i (I–J) antibodies. In (I), the upper 30 kDa band is the β5i containing the pro-peptide. The bar graphs represent the quantification of fluorescent signal intensity normalized to Vinculin. For β5i both bands were taken into consideration in the quantification of protein levels. Vinculin was used as the loading control (n = 4 per group). Values are mean ± SEM in arbitrary units.

To elucidate whether the observed decrease in the proteasome proteolytic activity of 26 S in LP is due to decreased gene expression, we have performed Real-Time qPCR to quantify the transcript levels of the subunits of 19 S and 20 S. Because the 20 S proteasome can also associate with an 11S regulator (PA28) that can modulate proteolytic activity of the 26 S complex, we also examined the transcript levels of the PA28α subunit of the 11S/PA28 regulator. There were no significant differences in the transcript levels of these subunits neither with pregnancy nor up to one week after delivery (in PP1 and PP7, Fig. 4). Western Blot analysis also revealed no significant differences in the expression levels of RPN2 and RPT4 (subunits of 19 S, Fig. 5A–D), in α7 (a subunit of 20S, Fig. 5E–F), in PA28α (Fig. 5G–H) nor in β5i (Fig. 5I–J) with pregnancy or after partum in the four groups mentioned above. For β5i, the two bands labeled with 26kDa and 30kDa, correspond to β5i and β5i containing its pro-peptide, respectively (Fig. 5I). Both bands were taken into consideration in the quantification of protein levels (Fig. 5J). Quantification of either band independent of the other also showed no difference between groups (data not shown).

### Pregnancy is Associated with Increased Nuclear Labeling of 20S core and 19S RPT4 in Cardiomyocytes

**Figure 6:**
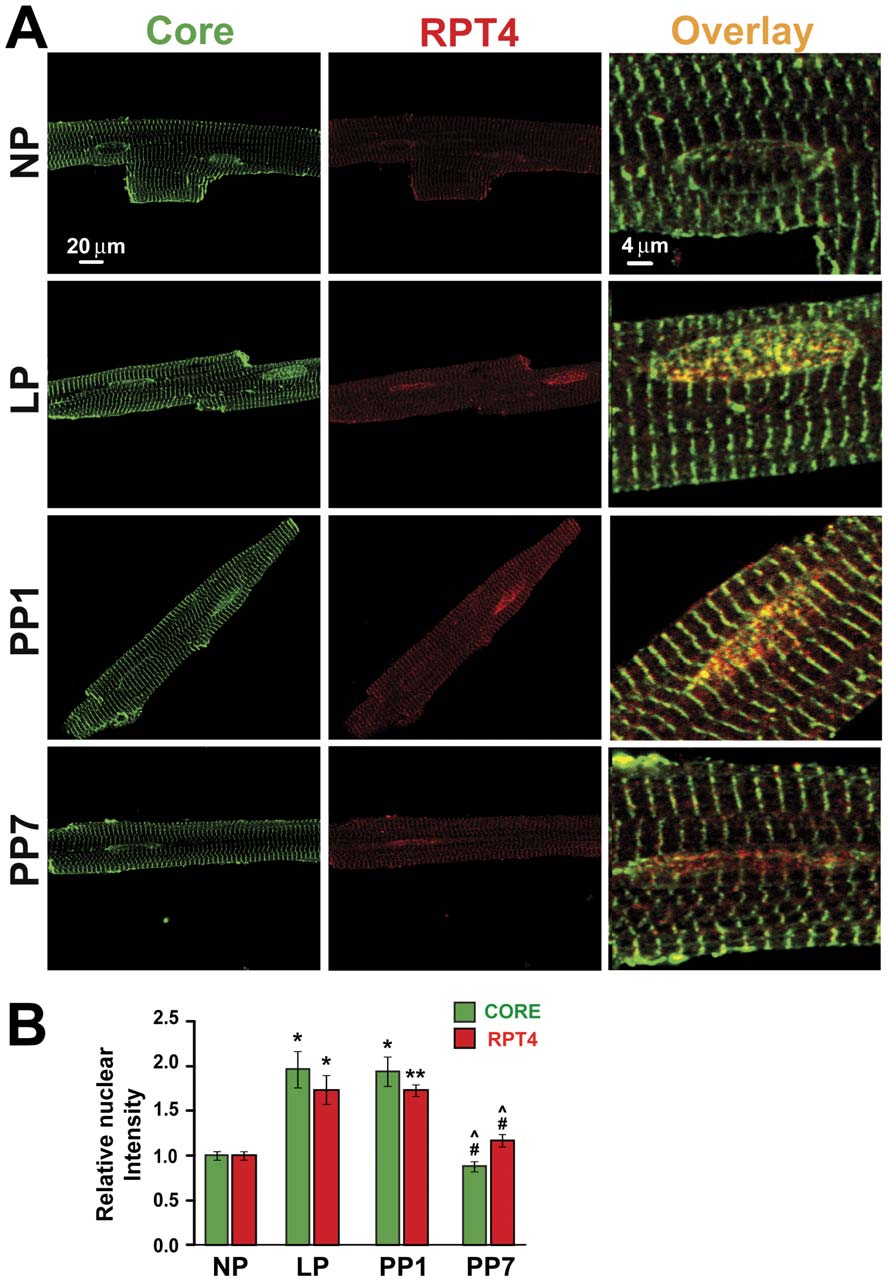
Increased nuclear labeling of core Subunits and RPT4 in late pregnancy was reversed one week postpartum. A. Representative single confocal sections of cardiomyocytes dissociated from non pregnant (NP), late pregnant (LP), one day post-partum (PP1) and seven days post-partum (PP7) are co-immunostained with anti-core (green) and -RPT4 (red) antibodies. The nuclear overlay of core and RPT4 are also shown at higher resolution. These results are representative of the labeling pattern observed in myocytes from 3 different animals in each group. B. Quantification of nuclear fluorescence labeling in the four groups mentioned above for core (green bars) and RPT4 (red bars) from at least 20–25 cells per group (n = 3 mice/group). Only the nucleus in the confocal plane of focus was taken into account. **denotes *p*<0.001 *vs.* NP, #*p*<0.05 *vs.* LP and ^∧^ *p*<0.05 *vs.* PP1.

To examine whether there are any changes in the subcellular distribution of proteasomal subunits in cardiomyocytes in LP, we have performed high resolution confocal microscopy. Fig. 6A shows typical examples of high resolution confocal images of cardiomyocytes co-labeled with anti-core (an antibody which recognizes six 20S subunits: α5/7, β1, β5, β5i, and β7) and anti-RPT4 (a subunit of 19S) antibodies. In NP, the vast majority of the stained proteins resided in the t-tubules and the nuclear labeling was very weak (Fig. 6A). In late pregnancy however, there is increased nuclear localization of both core and RPT4, which was reversed one week after parturition. In fact, quantification of the nuclear labeling revealed approximately a 2-fold upregulation of nuclear labeling with pregnancy (from 1±0.04 to 1.97±0.20 for core and from 1±0.04 to 1.73±0.16 for RPT4). The increased nuclear labeling of both proteins was sustained one day post partum (1.95±0.17 for core and 1.73±0.06 for RPT4) and was only reversed back to NP levels seven days after delivery (to 0.88±0.06 for core and 1.15±0.07 for RPT4, Fig. 6B).

### Pregnancy is Associated with Decreased Production of Reactive Oxygen Species

**Figure 7:**
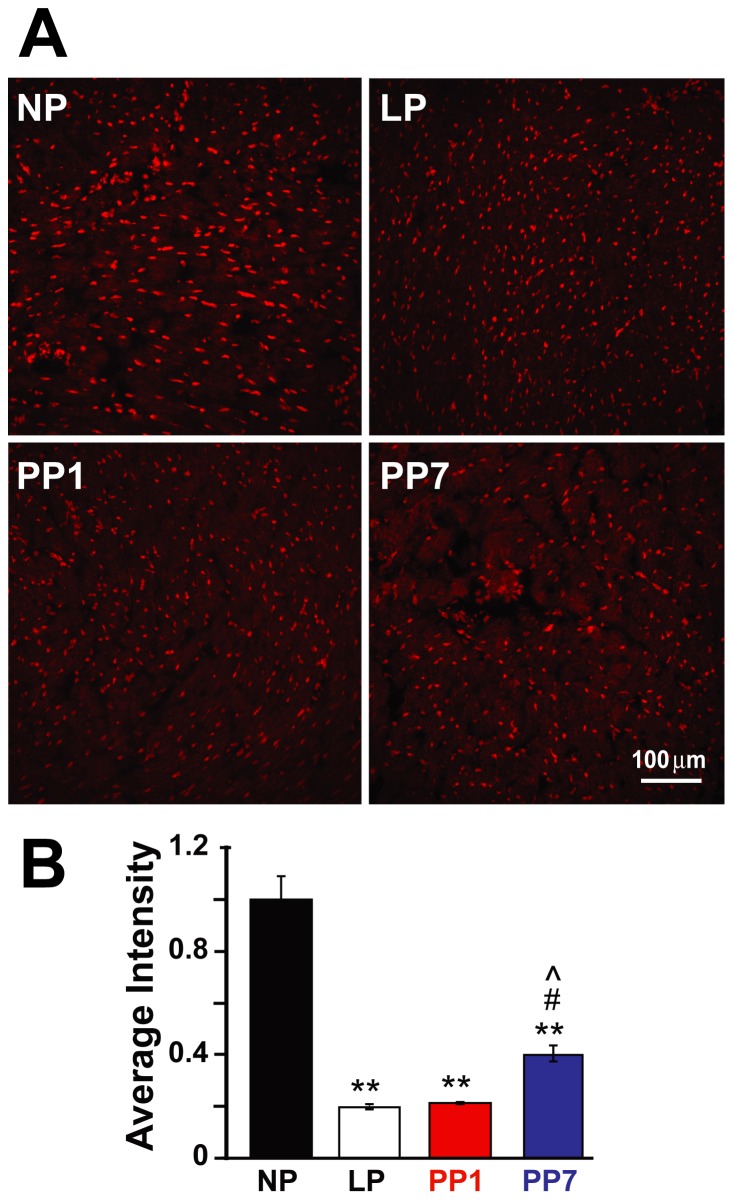
Superoxide production is decreased with pregnancy and remains low seven days after parturition. A. Representative dihydroethidium (DHE) staining of transverse heart sections in non pregnant (NP), late pregnant (LP), 1 day post-partum (PP1) and 7 days post-partum (PP7). Red staining indicates the presence of reactive oxygen species (ROS). B. Quantification of the DHE staining for detection of ROS in non pregnant (NP, black bar), late pregnant (LP, white bar), 1 day post-partum (PP1, grey bar) and 7 days post-partum (PP7, shaded bar). Values are mean ± SEM as normalized to NP (n = 3 per group), and **denotes *p*<0.001 *vs.* NP, #*p*<0.05 *vs.* LP and ^∧^ *p*<0.05 *vs.* PP1.

Since the nuclear proteasome selectively degrades oxidatively-damaged histones in the nuclei of mammalian cells [16], we performed a qualitative dihydroethidium (DHE) staining of cardiac cross-sections to assess the levels of reactive oxygen species production. We have found that late pregnancy is associated with decreased levels of reactive oxygen species compared to NP, and these levels remain low up to one week after parturition (Fig. 7), as the average intensity of DHE staining normalized to NP levels is reduced about 5-fold and partially recovers only seven days post-partum (0.198±0.010 in LP, 0.213±0004 in PP1, and 0.405±0.030 in PP7, normalized to NP).

### Pregnancy is Associated with Decreased Levels of Ubiquitinated Proteins

**Figure 8:**
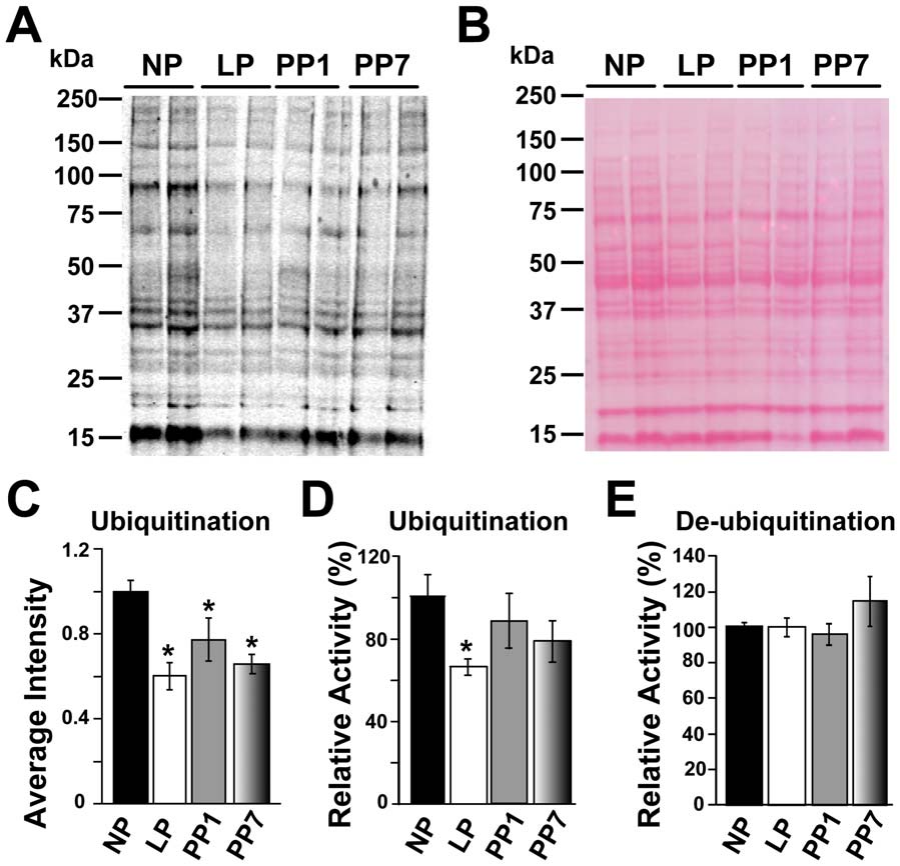
Pregnancy is associated with decreased polyubiquitinated protein levels, but not de-ubiqutination levels. A. Representative Western Blot of polyubiquitinated proteins (using the FK2 antibody) in whole heart lysates (100 µg) from non pregnant (NP), late pregnant (LP), 1 day post-partum (PP1) and 7 days post-partum (PP7). B. PonceauS was used as the loading control (n = 4 per group). C. Quantification of the polyubiquitinated proteins by Western Blot in non-pregnant (NP, black bar), late pregnant (LP, white bar), 1 day post-partum (PP1, grey bar) and 7 days post-partum (PP7, shaded bar). D. Polyubiquitination levels in NP, LP, PP1 and PP7 as determined by ELISA (using the FK1 antibody). E. De-ubiquitination activity levels in NP, LP, PP1 and PP7. Values are mean ± SEM and are normalized to NP, n = 4 per group and ^*^ denotes *p*<0.05 *vs.* NP.

To further elucidate the underlying mechanism for the observed decrease of 26S proteasome activity in late pregnancy, we quantified polyubiquitinated proteins in the heart using two different methods. Western Blot analysis against mono- and polyubiquitinated proteins revealed that ubiquitinated protein levels are significantly decreased in late pregnancy from 1±0.05 in NP to 0.60±0.06 in LP, and they remain low 7 days post-partum (0.65±0.04, in arbitrary units normalized to NP, Fig. 8A–C). Reduced polyubiquitinated protein levels in LP were also confirmed by ELISA: the relative amount was significantly downregulated from 100±10.41% in NP to 66.68±3.69% in LP (Fig. 8D). In order to examine whether the observed decrease in the levels of ubiquitinated proteins is not due to an increase in de-ubiquitination activity, we have also performed a de-ubiquitination assay and found that there are no significant changes in this system with pregnancy and parturition (Fig. 8E).

## Discussion

Characterization of cardiac physiological hypertrophy during pregnancy [15] led us to speculate that the late pregnant heart is “a better functioning heart,” as contractile efficiency and capacity is enhanced in response to increased force and stretch demand [17]. Here we show for the first time that the activity of the total 26S ATP-dependent proteasome, polyubiquitinated protein levels as well as the production of reactive oxygen species are reduced at the end of pregnancy, all of which may support our previous suggestion that a late pregnant heart is a better functioning heart. We also found that the activity of the 26S ATP-dependent proteasome was decreased in late pregnant hearts.

### Proteasome Activity and Cardiac Hypertrophy

The heart is the only organ in the body that is constantly bearing a heavy workload and a high metabolic rate. As such, it is essential that cardiac cells maintain a very efficient and tightly controlled system for removal of misfolded or damaged proteins [18]. During cardiac hypertrophy, the increased protein synthesis in cardiomyocytes could potentially result in an increase of misfolded or aberrant proteins. An increase in 26S proteasomal degradation could result in the clearance of these aberrantly folded proteins. Alternatively, an increase in protein degradation by the proteasome could lead to tissue atrophy. Although decreased proteasome activities have been shown during the progression of cardiac dysfunction [19], many studies report increased proteasome activity in compensated heart hypertrophy induced by trans-aortic constriction (TAC) both in mouse and canine models [20], [21], while the proteasome inhibitor epoxomicin prevented the development of pre-existing hypertrophy and the further reduction in the ejection fraction [6], [19]. Both trypsin-like activity (β2) and chymotrypsin-like activities (β5) were significantly increased in the subendocarium, which is subjected to the highest level of wall stress in a canine model of left-ventricular hypertrophy [20], [21]. In fact, increased proteasome activity has been suggested to be required for the development of compensated heart hypertrophy [20], [21]. Although during pregnancy the heart also develops compensated hypertrophy, the proteasome activity in this unique model of hypertrophy is not increased. In fact, the activity of the β1 and β2 subunits of the 26S proteasome is decreased in the LP heart. The decrease in 26S proteasome activity was not reflected by any changes in the inducible β5i subunit or in the PA28α subunit of the 11S/PA28 regulator. In contrast, in isoproterenol-induced cardiac hypertrophy, Drews et al. showed enhanced 26S proteasome activities, with a concomitant significant decrease in the caspase-like and trypsin-like 20S activities that may be due to a switch in proteasome subpopulations, altered expression and incorporation of the inducible β subunits [12].

### Expression of Proteasome Subunits and Cardiac Hypertrophy

Some controversy regarding the expression of proteasome subunits at the mRNA and protein levels exists. Most reports show an increase in 26S proteasome expression in different models of cardiomyopathy and hypertrophy. Otsuka et al. report an increase in 26S proteasome expression in patients suffering from dilated cardiomyopathy, possibly due to a compensatory mechanism in response to impaired proteasome activity [22]. Increased expression of the representative subunits of 19S (RPN2 and RPT11) and 20S (α6) have also been reported in the subendocarium of the canine model of left ventricular hypertrophy [20]. However, transcript levels of representative 20S subunits have been shown to be decreased in failing hearts [19], suggesting possible post-translational modifications. Here we did not observe any significant differences in the transcript or protein levels of α7 (a subunit of 20S), RPN2 and RPT4 (subunits of 19S) the regulatory subunit PA28α and the inducible subunit β5i (protein levels only) with pregnancy (Figs. 4, 5), further suggesting that pregnancy-induced hypertrophy has a molecular signature unlike all other models of hypertrophy that have previously been studied.

### Pregnancy is Associated with Decreased Levels of Ubiquitinated Proteins

Proteins that are targeted for degradation by the proteasome must first initially be covalently tagged with ubiquitin molecules by the E2–E3 ligase complex [9]. These ubiquitin molecules are then recognized by the 19S regulatory particle of the 26S proteasome complex in an ATP-dependent binding [23]. Therefore, protein ubiquitination is one of the key mechanisms for targeting a peptide to be degraded by the proteasome’s proteolytic pathway, and ubiqutination levels are also important for the proteasome activity. Here, we have found that pregnancy is associated with decreased ubiquitinated protein levels (Fig. 8). Two independent methods (Western Blot and ELISA) were utilized to show that the levels of ubiquitination in the heart were decreased in late pregnancy relative to non pregnant mice. However, unlike pregnancy, immunocytochemical experiments previously revealed markedly increased expression levels of ubiquitin in patients with decompensated cardiomyopathy [22]. Increased ubiqutination levels have also been reported in experimental models of pressure overload-induced left ventricular hypertrophy in murine and canine hearts [18], [19], [20]. Lower proteasome activity may conserve energy as less ATP would be needed for protein unfolding by the 19S complex and this may be beneficial to the heart. Changes in protein ubiquitination can occur from changes in proteasome activity, changes in de-ubiquitination activity or changes in the ubiquitin-conjugating activity system (E1, E2 and E3 enzymes). Lower proteasome activities are unlikely to cause lower polyubiquitination levels since the proteasome readily degrades polyubiquitinated proteins. Investigation of the de-ubiquitination activity showed that the total de-ubiquitination activity was not significantly affected by pregnancy. These results suggest that the ubiquitin-conjugating activity system may be lowered by pregnancy. It is possible that the proteasome has potentially less substrates (less polyubiquitinated proteins) to degrade post-translational modifications (which are known to affect the activity of the proteasome [13], [14]), which may be responsible for the reduced activity of the proteasome since the proteasomal gene and protein expression seems to be unchanged.

### Reactive Oxygen Species Production and the 26S Proteasome

The proteasomal system has previously been shown to be the major proteolytic system involved in the removal of oxidized proteins, with the 26S proteasome being the most sensitive to oxidative stress [16]. Although the 26S proteasome generally functions as part of the ubiquitin-proteasome pathway, it also has the capacity to degrade certain unfolded or damaged proteins, including “aged”, denatured proteins, or proteins that have been oxidatively damaged, without initial ubiquitin tagging [13], [24]. We observed that the reactive oxygen species (ROS) generated in pregnancy is decreased and remains low one week after delivery (Fig. 6). ROS has been previously shown to increase the ubiquitin-conjugating activity and expression of genes for E3 enzymes (MuRF1 and MaFbx) in skeletal muscle myotubes [25]. Lower levels of ROS may account for the decreased polyubiquitination levels in the LP hearts.

### Hormones and Proteasome Activity

It is also possible that estrogen may affect the proteasome, as interferon-induced oxidative stress has been shown to be associated with decreased proteasome amount and increased polyubiquitination [26]. Hormones, either directly or via the control of the metabolic status, can affect the ubiquitin-mediated control of protein degradation, as glucocorticoids have previously been shown to cause catabolic protein breakdown [27]. In skeletal muscle, degradation of cell proteins is of major physiological importance, and the size of a muscle cell is tightly regulated by the overall rate of proteolysis, a process precisely regulated by hormones and cytokines [28]. Lastly, Genistein, a soy isoflavone with affinity for the estrogen receptor beta, has been shown to inhibit 20S proteasome activity in human prostate cancer cells [29]. The level of estrogen drastically increases at the end of pregnancy, but it is not clear whether estrogen treatment could regulate proteasome activity in the heart. Here we report for the first time that estrogen did not have any effect on the three proteolytic activities of the murine cardiac 26S proteasome. Thus, the changes in proteasome activity occurring in pregnancy cannot be attributed to the surge of estrogen.

Taken together, our results suggest that the ubiquitination, proteasome proteolytic pathway and the production of reactive oxygen species are affected by pregnancy. Late pregnancy is associated with a decrease in the polyubiquitination levels, which could be explained at least in part by reduced ROS production.
